# APOE4 impairs macrophage lipophagy and promotes demyelination of spiral ganglion neurons in mouse cochleae

**DOI:** 10.1038/s41420-025-02454-4

**Published:** 2025-04-21

**Authors:** Junru Chen, Haibing Chen, Qinjun Wei, Yajie Lu, Tianming Wang, Xiuhong Pang, Guangqian Xing, Zhibin Chen, Xin Cao, Jun Yao

**Affiliations:** 1 Department of Medical Genetics, School of Basic Medical Science, Nanjing Medical University, Nanjing, China; 2 Department of Otolaryngology, the First Affiliated Hospital with Nanjing Medical University, Nanjing, China; 3 Jiangsu Key Laboratory of Xenotransplantation, Nanjing Medical University, Nanjing, China; 4 Central Laboratory, Translational Medicine Research Center, the affiliated Jiangning Hospital of Nanjing Medical University, Nanjing, China; 5 Department of Otolaryngology-Head and Neck Surgery, the Affiliated Taizhou People’s Hospital of Nanjing Medical University, Taizhou School of Clinical Medicine, Nanjing Medical University, Taizhou, China

**Keywords:** Autophagy, Gene regulation

## Abstract

The *ApoE-ε4* gene is a well-established genetic risk factor for neurodegenerative diseases, such as Alzheimer’s disease and multiple sclerosis, which are characterized by axonal demyelination in the central nervous system. Recent studies have implicated *ApoE-ε4* in age-related hearing loss (ARHL), suggesting a potential role of APOE4 isoform in peripheral nervous system degeneration. However, the role of APOE4 in ARHL are still unclear. In this study, we explored the potential role of APOE4 in axonal demyelination of spiral ganglion neurons (SGNs). *ApoE-ε4/ε4* (APOE4) and *ApoE-ε3/ε3* (APOE3) mice were used to characterize SGNs. The effect of APOE4 on phagocytosis and autophagy as well as intracellular cholesterol level was evaluated in resident cochlear macrophages (RCMs) and mouse bone marrow-derived macrophages (BMDMs). The results showed that significant axonal demyelination was observed in SGNs of 10-month-old APOE4 mice, accompanied by the presence of myelin debris engulfed by RCMs. Meanwhile, inhibited phagocytosis of myelin debris and impaired lipophagy were detected in APOE4 RCMs and APOE4 BMDMs with an aberrant accumulation of lipid droplets (LDs), which could be reversed by trehalose treatment. This study provided a deep insight into the pathogenesis of APOE4-induced axonal demyelination of SGNs associated with the impaired lipophagy in RCMs, which helped to elucidate the underlying mechanism of *ApoE-ε4* in ARHL.

## Introduction

Apolipoprotein E (APOE), a lipid and cholesterol transporter, is critical for maintaining cholesterol homoeostasis and facilitating neuronal plasticity. APOE isoforms are encoded by three allelic genotypes: *ApoE-ε2*, *ApoE-ε3*, and *ApoE-ε4* [1]. Notably, the *ApoE-ε4* allele has been identified as a significant risk factor for conditions such as Alzheimer’s disease (AD) [2, 3] and multiple sclerosis (MS) [4], with a dose-dependent effect on both the risk and age of onset.

Emerging evidence has also linked the *ApoE-ε4* allele to age-related hearing loss (ARHL), a prevalent condition characterised by the progressive deterioration of auditory function associated with aging [5]. A recent large cohort study reported a two-fold increase in the risk of ARHL for individuals carrying the *ApoE-ε4* allele, with the most pronounced hearing loss observed in homozygous individuals [6]. The pathophysiology of ARHL involves the loss of auditory sensory cells and dysfunction of the auditory nervous system. Spiral ganglion neurons (SGNs) are crucial for the transmission of auditory signals to the brain, and their degeneration is a primary contributor to ARHL. Previous studies have demonstrated involvement of both acute and progressive changes in the ultrastructure of SGN axons in sensorineural hearing loss [7, 8]. Myelin sheaths, crucial for the normal function of axons, are lipid membranes that wrap around neuronal axons and are primarily composed of myelin. These myelin sheaths primarily serve as insulation, ensuring accurate and efficient transmission of electrical signals [9, 10]. Axonal demyelination is a common hallmark of neurodegenerative diseases [11, 12]. Therefore, the structural and functional integrity of myelin sheaths is crucial for the function of SGNs.

Emerging evidence has increasingly implicated APOE4 in the pathogenesis of demyelination within the corpus callosum derived from APOE4 mice [13] and in induced pluripotent stem cells [14], albeit the molecular mechanisms remain to be fully elucidated. APOE isoforms, notably expressed in neuronal and microglial populations, function as pivotal cholesterol transporters, modulating lipid efflux and intracellular processes [15]. In AD patients carrying the *ApoE-ε4* allele, abnormal accumulation of lipid droplets (LDs) in neurons and microglia has been detected [16, 17]. This phenomenon may be intricately linked to the modulatory effects of APOE4 on microglial phagocytosis, the disruption of cholesterol homoeostasis, and autophagic processes. Some studies report a dampened phagocytic efficacy in APOE4 human microglia-like cells [18, 19], posing a challenge to myelin repair. While the negative influence of APOE4 on microglial cholesterol metabolism is recognised, its effect on microglial autophagy is still under scrutiny.

In the peripheral nervous system, macrophages are also crucial for maintaining neuronal homoeostasis and coordinating inflammatory responses to neuronal injury [20]. Reduced clearance of myelin debris by macrophages may lead to excessive accumulation of myelin debris, which compromises the rate of myelin regeneration and quality of tissue repair [21, 22]. Additionally, the defect autophagy of macrophage leads to continuous accumulation of LDs and disrupts cellular lipid homoeostasis [23]. Lipophagy, a specialised form of autophagy targeting LDs, is implicated in the degradation of myelin debris. Deficit lipophagy may lead to the accumulation of LDs, inflammatory activation and impaired myelin regeneration, further exacerbating neurodegenerative processes [24, 25].

In this study, the role of APOE4 in the axonal demyelination of SGNs associated with lipophagy in resident cochlear macrophages (RCMs) were investigated to gain insights into the pathogenic mechanism of APOE4 in ARHL.

## Results

### APOE4 promotes demyelination of mouse cochlear SGNs

**Fig. 1:**
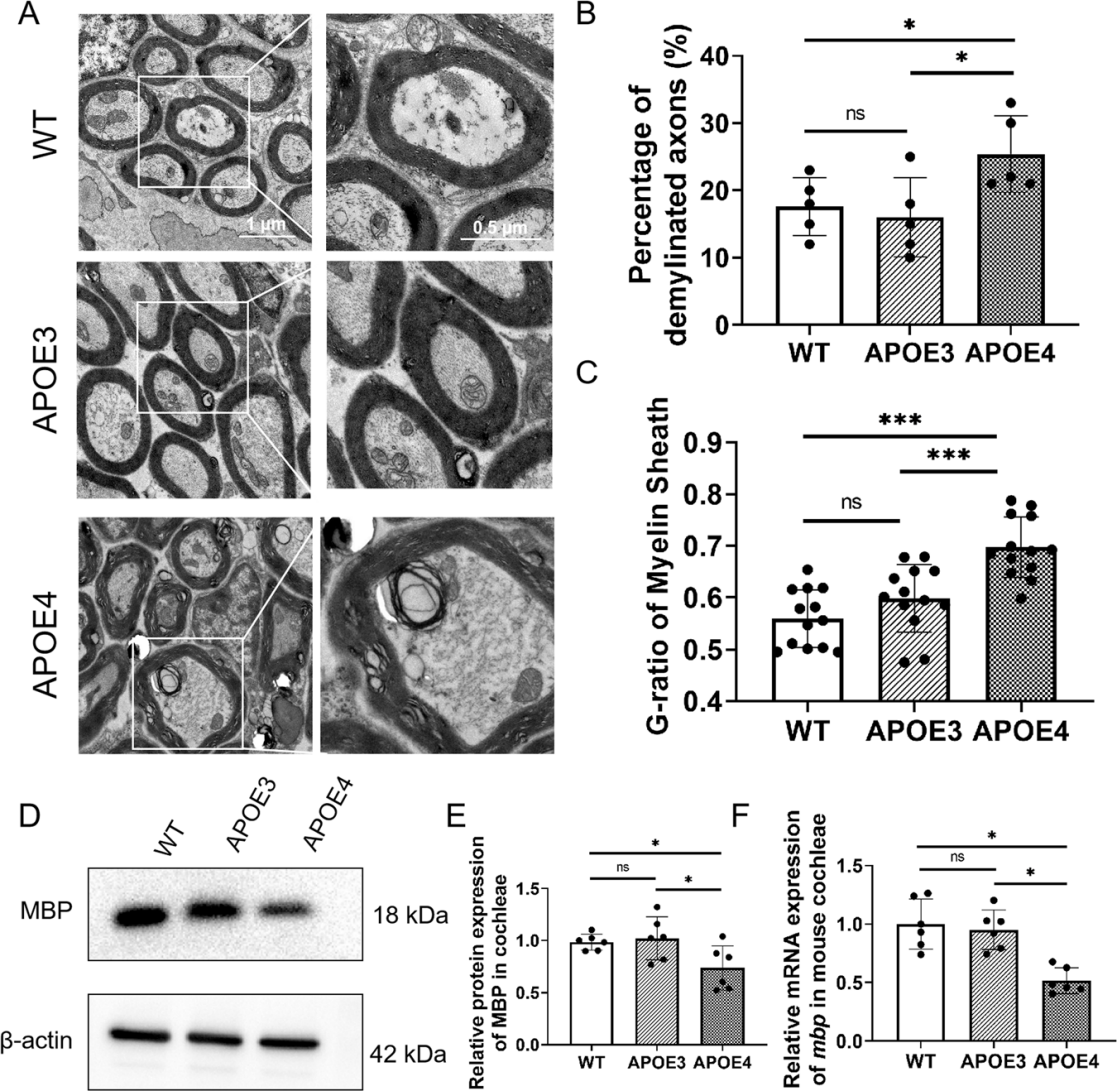
Structural characterization of SGN myelin in the cochleae of 10-month-old APOE3 and APOE4 mice. **A** TEM images of the SGN ultrastructure in the cochleae of WT, APOE3 and APOE4 mice. Scale bar: 1 μm. Solid frames denote the enlarged region showing axonal demyelination (APOE4) and compact myelin (APOE3). Scale bar: 0.5 μm. **B** Percentage of demyelinated SGNs in the cochleae of WT, APOE3 and APOE4 mice (20 SGNs in every view, each dot represents all SGNs in a microscope field, *n* = 5; ns not significant, *P < 0.05 by one-way ANOVA). **C** Myelin g-ratios in the SGNs of WT, APOE3 and APOE4 mice (20 SGNs in every view, each dot represents all SGNs in a microscope field, *n* = 13; ns not significant, ***P < 0.001 by one-way ANOVA). G-ratio in the APOE4 SGNs was lower than that in WT and APOE3 controls. **D** MBP protein expression in the cochleae of WT, APOE3 and APOE4 mice. The expression of MBP was significantly downregulated in the cochleae of APOE4 mice compared with WT and APOE3 controls. **E** Relative protein expression of MBP in the cochleae of WT, APOE3 and APOE4 mice (*n* = 6; ns not significant, *P < 0.05 by one-way ANOVA). **F** Relative mRNA expression of MBP in the cochleae of WT, APOE3 and APOE4 mice (*n* = 6; ns not significant, *P < 0.05 by one-way ANOVA).

Peripheral fibres of SGNs afferents in 10-month-old mouse cochleae were observed by transmission electron microscopy (TEM) (Fig. 1A). In wild-type (WT) and *ApoE-ε3/ε3* (APOE3) mice, myelin sheaths typically manifested as tightly wrapped multilamellar membrane around axons, with a clear periodic structure that facilitated rapid conduction of neural impulses. In contrast, in *ApoE-ε4/ε4* (APOE4) mice, the incompact myelin sheaths were observed surrounding the spiral ganglion neurons (SGNs) (Fig. 1A). The proportion of demyelinated SGNs in APOE4 mice is higher than that in WT and APOE3 mice (Fig. 1B). The myelin g-ratio, which is the ratio of the inner to the outer diameter of the myelin sheath, also indicates that the myelin sheaths in APOE4 mice have thinner walls than that in WT and APOE3 mice (Fig. 1C).

In addition, myelin basic protein (MBP), one of the main proteins in the myelin sheath, was detected to be downregulated at both the mRNA and protein levels in whole cochleae of 10-month-old APOE4 mice, (Fig. 1D–F), which was consistent with the significant demyelination of SGNs. However, there were no significant differences in the morphology of myelin sheaths in SGNs of WT and APOE3 mice, nor were there significant differences in the expression of MBP.

### APOE4 inhibited phagocytosis of myelin debris by RCMs

**Fig. 2:**
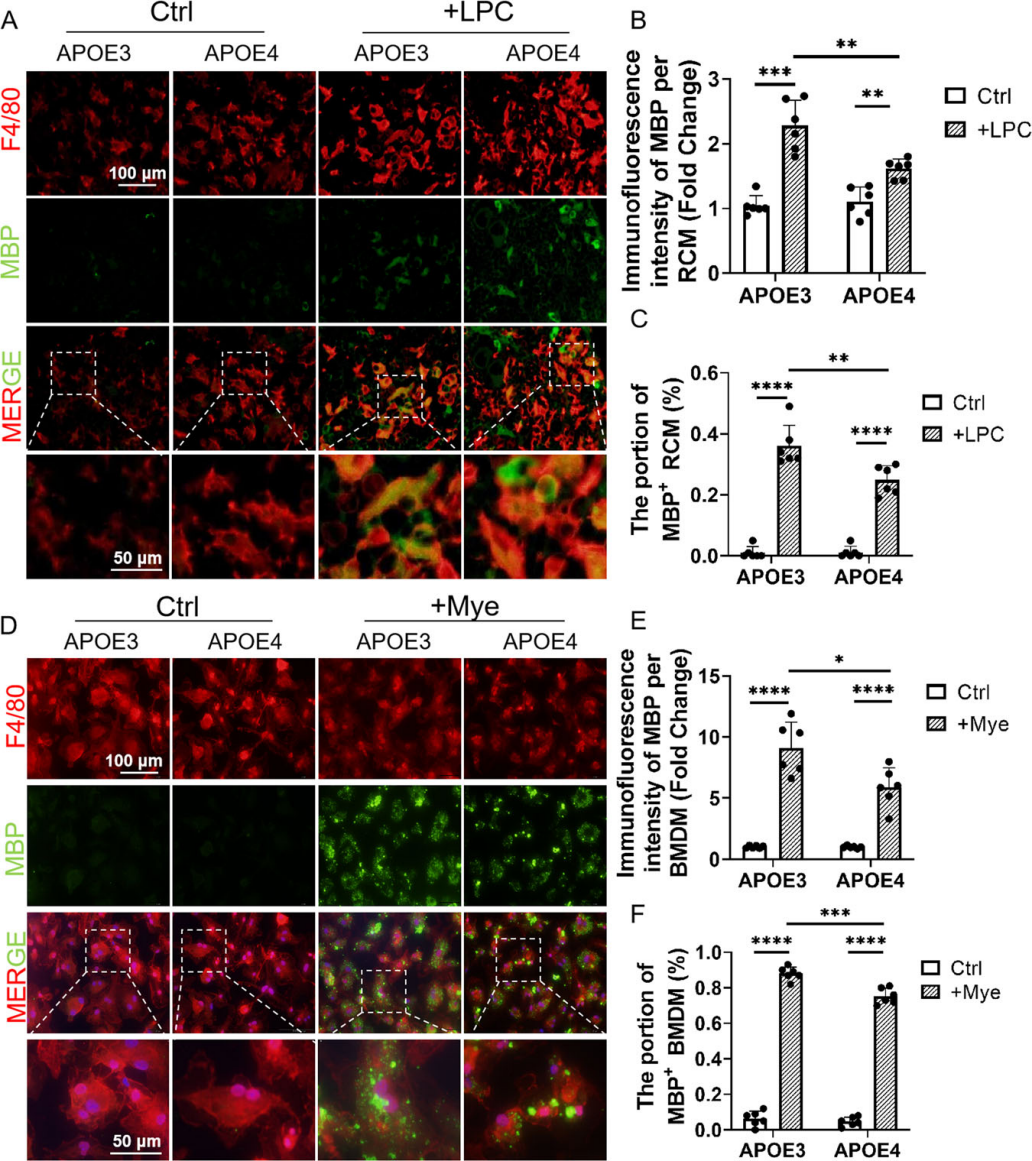
APOE4 impairs the phagocytic ability in RCMs and BMDMs. **A** Immunofluorescent assay of RCMs labelled with MBP (green) and F4/80 (red) in the cochleae of 10-month-old APOE4 and APOE3 mice. Scale bar: 100 μm. Dashed frames denote the enlarged region showing the colocalization of MBP and F4/80. Scale bar: 50 μm. **B** Quantification of MBP fluorescence intensity per cell (20 RCMs in every view, each dot represents all RCMs in a microscope field, *n* = 6; **P < 0.01, ***P < 0.001 by two-way ANOVA). **C** The proportion of MBP^+^ RCMs (20 RCMs in every view, each dot represents all RCMs in a microscope field, *n* = 6; **P < 0.01, ****P < 0.0001 by two-way ANOVA). **D** Immunofluorescent assay of APOE3 and APOE4 BMDMs labelled with MBP (green) and F4/80 (red). Scale bar: 100 μm. Dashed frames denote the enlarged region showing the colocalization of MBP and F4/80. Scale bar: 50 μm. **E** Quantification of MBP fluorescence intensity per cell (50 BMDMs in every view, each dot represents all BMDMs in a microscope field, *n* = 6; *P < 0.05, ****P < 0.0001 by two-way ANOVA). **F** The proportion of MBP-positive (MBP^+^) BMDMs (50 BMDMs in every view, each dot represents all BMDMs in a microscope field, *n* = 6; ***P < 0.001, ****P < 0.0001 by two-way ANOVA).

In previous studies, macrophages were thought to be recruited from other locations after acute or chronic demyelinating injury to engulf shed myelin debris and help to repair neuron damage [21, 26]. To investigate the phagocytosis of myelin debris by RCMs, lysophosphatidylcholine (LPC), a rapidly demyelinating drug, was injected through the mouse tympanic membrane [27]. Immunohistochemical assay showed that LPC-treatment induced the decreased MBP expression and increased F4/80-positive cells, indicating demyelination of SGNs (Supplementary Fig. S1A, B). Immunofluorescent assay showed that MBP was co-localised with F4/80-labelled RCMs in the cochleae of LPC-treated APOE4 and APOE3 mice (Fig. 2A), indicating that shed myelin debris was phagocytosed by RCMs. Moreover, a lower proportion of MBP-positive RCMs with reduced fluorescence intensity of MBP was detected in the cochleae of LPC-treated APOE4 mice (Fig. 2B, C).

RCMs in mature mice are known to be constantly replaced by monocytes produced by myeloid progenitors derived from bone marrow, and monocytes differentiate into macrophages under cytokine stimulation [28]. Bone marrow derived macrophages (BMDMs) is a well-established in vitro model for understanding the mechanisms controlling polarization of activated macrophages [23]. By flow cytometry, BMDMs and RCMs were sorted using CD45 and CD11b as the macrophage markers, and the results indicated that protein typing of RCMs (CD45^high^, CD11b^high^) were homologous to that of BMDMs (Supplementary Fig. S2) [29, 30]. Therefore, mouse BMDMs were used in subsequent in vitro experiments. Immunofluorescent staining showed that MBP was co-localised with F4/80-labelled BMDMs treated with myelin debris (Fig. 2D). It was noteworthy that the proportion of MBP-positive BMDMs as well as fluorescence intensity of MBP per BMDM was detected to be lower in APOE4 BMDMs than that in APOE3 controls (Fig. 2E, F). These results indicated that APOE4 diminished the phagocytic capacity of RCMs and BMDMs for the clearance of myelin debris.

### APOE4 impaired autophagy in BMDMs and RCMs

Proteomic Gene Ontology analysis showed that most differentially expressed proteins between APOE3 and APOE4 BMDMs were enriched as components of membrane-bound organelles, including lysosomes, and those related to autophagy were found to be generally downregulated (Supplementary Fig. S3A, B). Autophagy is an evolutionarily conserved catabolic process that eliminates harmful components through lysosomal degradation and maintains cellular homoeostasis, and autophagy dysfunction is implicated in pathological processes, including demyelinated lesions [31, 32] Lipophagy is an intracellular lysosomal-mediated autophagy process, which refers to the process by which intracellular lysosomes remove and degrade LDs. In demyelinating diseases, increasing the lipophagy in macrophages could promote myelin repair [23, 33, 34]. The proteomic analysis indicated that APOE4 could induce defect lipophagy of RCMs, impair the capacity of myelin debris clearance and thereby promote axonal demyelination of SGNs.

**Fig. 3:**
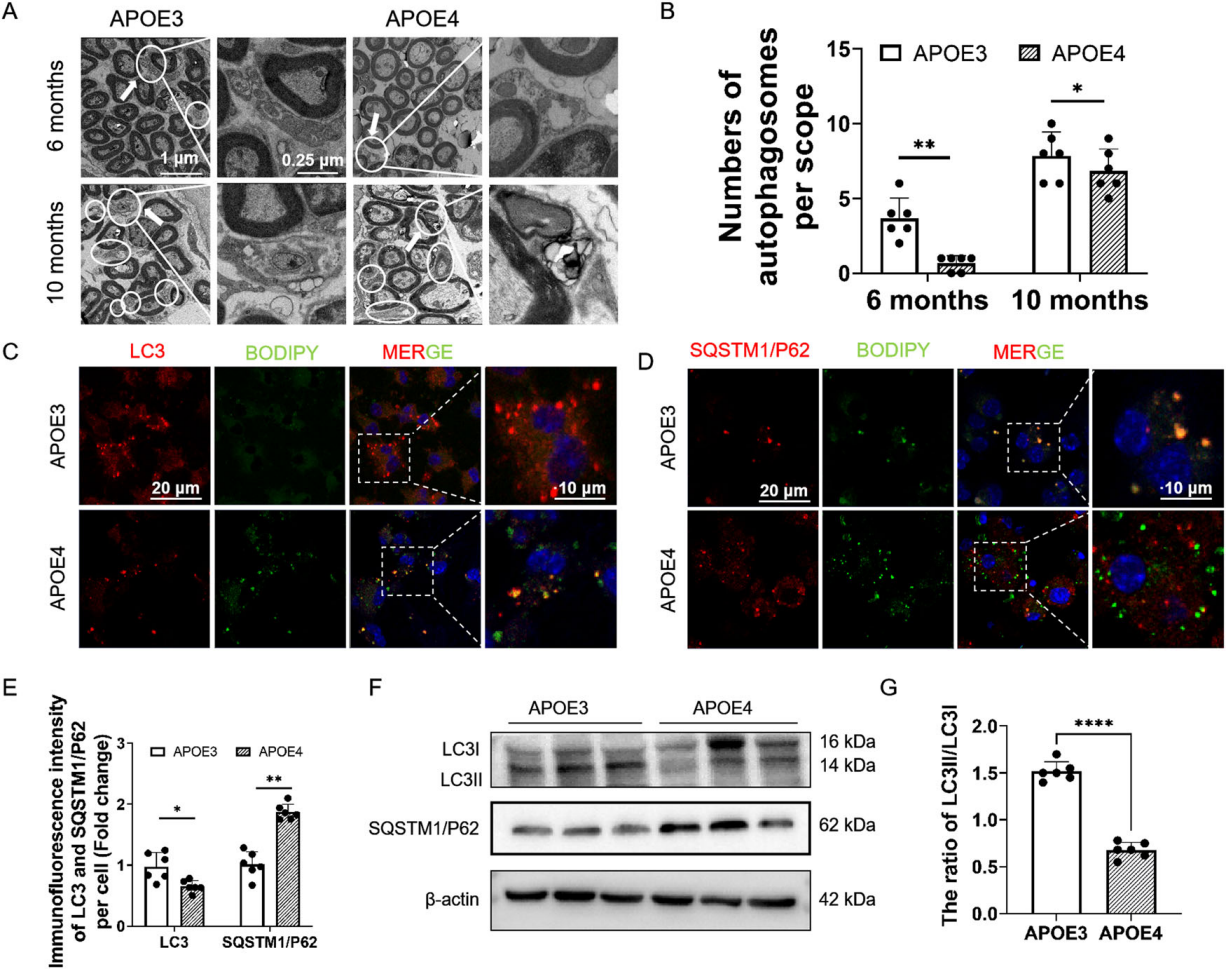
APOE4 induced the impairment of autophagy in RCMs and BMDMs. **A** TEM of RCM autophagosomes (Solid circle) near SGNs in cochleae of 6- and 10-month-old APOE4 and APOE3 mice. Scale bar: 1 μm. The arrows denote the enlarged region showing RCM autophagosomes. Scale bar: 0.25 μm. **B** Quantification of RCM autophagosomes per scope. (10 autophagosomes in every view, each dot represents all autophagosomes in a microscope field, *n* = 6; *P < 0.05, **P < 0.01 by one-way ANOVA). **C** Immunofluorescent staining of LDs (BODIPY in green) in APOE3 and APOE4 BMDMs (LC3 in red). Scale bar: 20 μm. The dashed frames denote the enlarged region showing BMDMs by merging BODIPY (green) and LC3 (red). Scale bar: 10 μm. **D** Immunofluorescent assay of LDs (BODIPY in green) in APOE3 and APOE4 BMDMs (SQSTM1 in red). Scale bar: 20 μm. The dashed frames denote the enlarged region showing BMDMs by merging BODIPY (green) and SQSTM1/P62 (red). Scale bar: 10 μm. **E** Fluorescence intensity of LC3 (panel **C**) and SQSTM1 (panel **D**) per cell of APOE3 and APOE4 BMDMs within a microscope field (10 BMDMs in every view, each dot represents all BMDMs in a microscope field, *n* = 6; *P < 0.05 by one-way ANOVA). **F** LC3 and SQSTM1/P62 protein expression in APOE3 and APOE4 BMDMs. Compared with APOE3 controls, the ratio of LC3II/LC3I was significantly decreased, and the expression of SQSTM1/P62 was significantly increased in APOE4 BMDMs. **G** The ratio of LC3II/LC3I in APOE3 and APOE4 BMDMs (*n* = 6; ****P < 0.0001 by one-way ANOVA).

TEM observations showed that RCMs containing autophagosomes were present near SGNs in the cochleae of APOE3 and APOE4 mice (Fig. 3A). The number of RCM autophagosomes per scope increased with age, but was less in APOE4 mice than in age-matched APOE3 mice (Fig. 3B). It was also observed that 10-month-old APOE4 mice showed significant demyelination of SGNs (Fig. 3A), which could be attributable to the impaired autophagy in APOE4 RCMs. To depict the effect of APOE4 on autophagy, LC3 and SQSTM1/p62 were determined as autophagy markers in BMDMs. During the autophagosomal degradation process, LC3I is converted to LC3II through lipidation, which increases the LC3II/LC3I ratio, and the reduction of LC3II to LC3I ratio and an increase of SQSTM1/p62 levels were considered to be associated with an impairment of autophagy [35]. Immunofluorescent staining showed that LC3 was significantly observed to be co-localized with BODIPY in APOE3 BMDMs (Fig. 3C). Meanwhile, LC3 was downregulated and SQSTM1/P62 was upregulated in APOE4 BMDMs (Fig. 3C–E). Western blot assay showed that the LC3II/LC3I ratio in APOE4 BMDMs was lower than that in APOE3 BMDMs, and high-expressed SQSTM1/p62 was detected in APOE4 BMDMs (Fig. 3F, G). SQSTM1/P62 was also found to be significantly upregulated in the cochleae of 10-month-old APOE4 mice (Supplementary Fig. S4). These results indicated that APOE4 impaired autophagy in BMDMs and RCMs.

### APOE4 induced cholesterol accumulation in BMDMs and RCMs

**Fig. 4:**
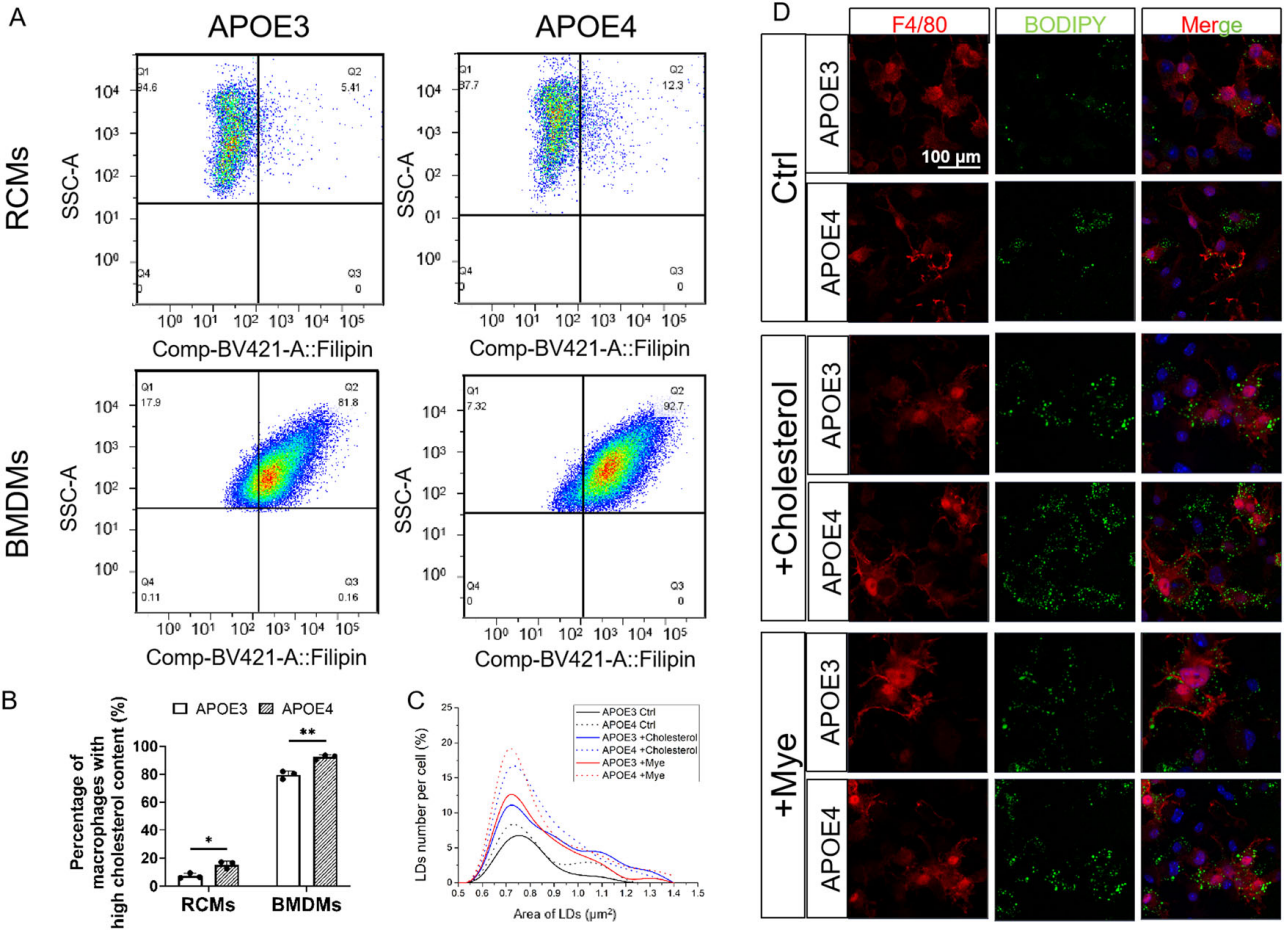
APOE4 disrupted cholesterol homoeostasis in RCMs and BMDMs. **A** Flow cytometry assay of cholesterol in APOE3/APOE4 RCMs and BMDMs. BMDMs and RCMs were stained using CD45^high^, CD11b^high^ and F4/80^+^ as the macrophage markers. **B** Percentage of RCMs and BMDMs with high cholesterol content (*n* = 3, *P < 0.05 by one-way ANOVA, BMDMs: *n* = 3, **P < 0.01 by one-way ANOVA). **C** Number of LDs per cell in APOE3 and APOE4 BMDMs untreated (Ctrl) or treated with cholesterol (+Cholesterol) and Myelin debris (+Mye) (*n* = 6). **D** Immunofluorescent assay of LDs (BODIPY in green) in APOE3 and APOE4 BMDMs (F4/80 in red) untreated (Ctrl) or treated with cholesterol (+Cholesterol) and Myelin debris (+Mye). Scale bar: 100 μm.

Lipophagy deficiency can cause pathological disruption of cholesterol efflux/homoeostasis in macrophages [23]. Proteomic Kyoto Encyclopedia of Genes and Genomes (KEGG) analysis indicated the disruption of lipid homoeostasis in APOE4 BMDMs (Supplementary Fig. S5A, B). To determine the intracellular cholesterol, APOE4 RCMs and APOE4 BMDMs were stained with Filipin-III, and were analysed by flow cytometry. Compared with APOE3 controls, an increase of cholesterol was detected in both APOE4 RCMs and APOE4 BMDMs (Fig. 4A, B). BODIPY staining of LDs was performed on the groups of untreated BMDMs, cholesterol-treated BMDMs and myelin-debris-treated BMDMs (Fig. 4C, D). In untreated groups, the number of LDs in APOE4 BMDMs was greater than that in APOE3 BMDMs. Interestingly, both the cholesterol treatment and myelin-debris treatment led to a more significant increase of LDs in APOE4 BMDMs than that in APOE3 BMDMs. It has been reported that the clearance of myelin debris required cholesterol transporters, including apolipoprotein E [15]. The above results indicated that APOE4 induced the defect lipophagy in BMDMs, which diminished the cholesterol efflux capacity of BMDMs. As a result, the treatment of cholesterol or cholesterol-rich myelin debris could disrupt cholesterol homoeostasis and lead to abnormal LDs accumulation in APOE4 BMDMs.

### Deficit autophagy in APOE4 BMDMs was rescued by trehalose treatment

**Fig. 5:**
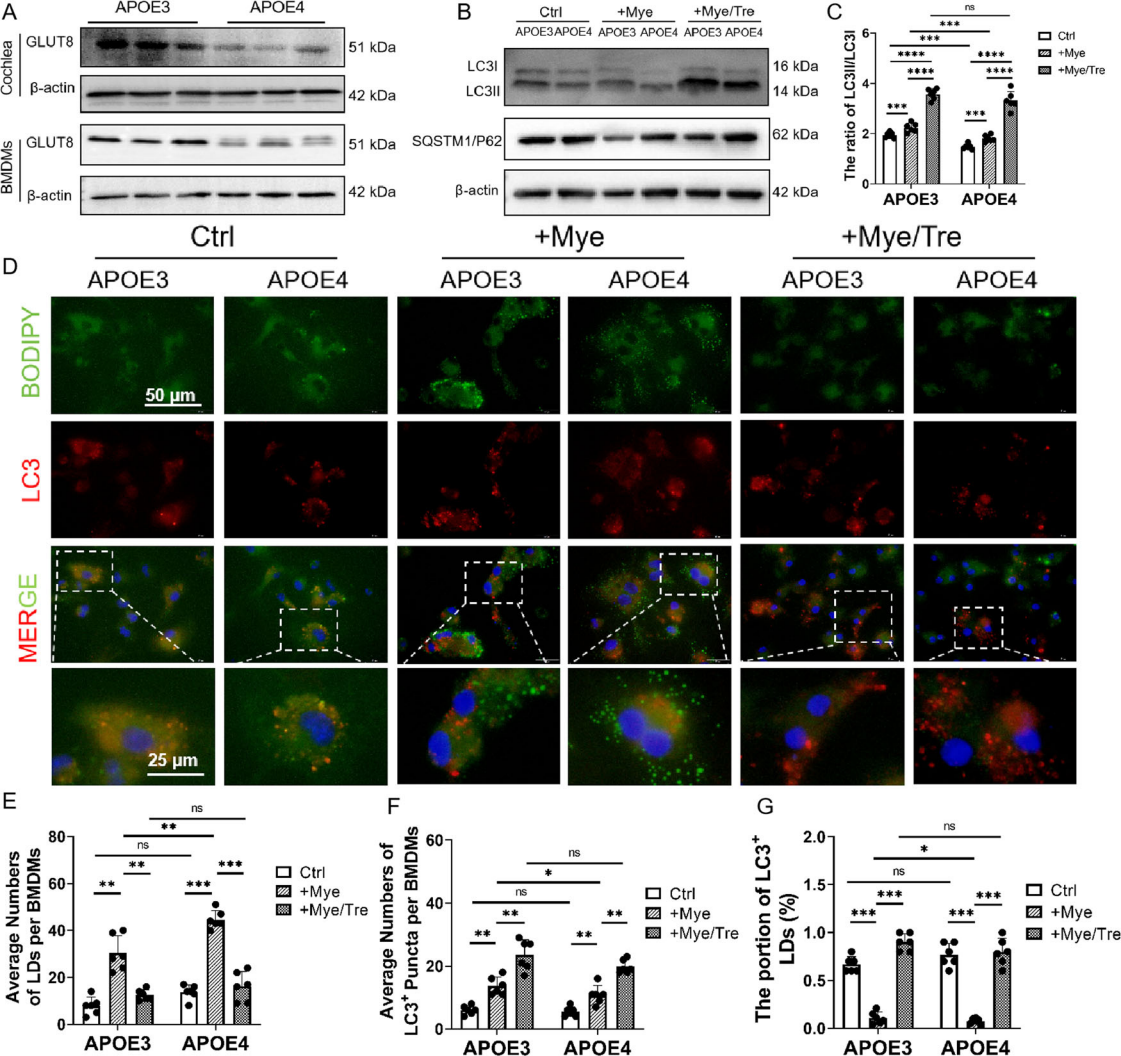
Impaired autophagy in APOE4 BMDMs was partially rescued by trehalose treatment. **A** Expression of GLUT8 in BMDMs and cochleae of 10-month-old APOE4 and APOE3 mice. The expression of GLUT8 was significantly decreased in APOE4 RCMs and BMDMs compared with APOE3 controls. **B** Expression of LC3 and SQSTM1/P62 in APOE3 and APOE4 BMDMs. Cells were treated with myelin debris (+Mye) or were co-treated with both myelin debris and trehalose (+Mye/Tre). The ratio of LC3II/LC3I was significantly decreased and SQSTM1/P62 was upregulated in trahalose-treated APOE4 and APOE3 BMDMs (+Mye), which could be reversed by the co-treatment of trehalose (+Mye/Tre). **C** The ratio of LC3II/LC3I in APOE3 and APOE4 BMDMs (*n* = 6, ns not significant, ***P < 0.001, ****P < 0.0001 by two-way ANOVA). **D** Immunofluorescence staining of LDs (BODIPY in green) in APOE3 and APOE4 BMDMs (LC3 in red). Scale bar: 50 μm. Dashed frames denote the enlarged region in the panel below. Scale bar: 25 μm. Cells were treated with myelin debris (+Mye) or were co-treated with both myelin debris and trehalose (+Mye/Tre). **E** Average number of LDs per BMDM (10 BMDMs in every view, each dot represents all BMDMs in a microscope field, *n* = 6; ns not significant, **P < 0.01, ***P < 0.001 by two-way ANOVA). **F** LC3-positive (LC3^+^) puncta per BMDM (10 BMDMs in every view, each dot represents all BMDMs in a microscope field, *n* = 6; ns not significant, *P < 0.05; **P < 0.01 by two-way ANOVA). **G** Portion of LC3-positive (LC3^+^) LDs in APOE4 and APOE3 BMDMs (100 LDs in every view, each dot represents all LDs in a microscope field, *n* = 6, ns not significant, *P < 0.05; ***P < 0.001 by two-way ANOVA).

Among the differentially expressed proteins, GLUT8 was downregulated, which indicated lipophagy deficiency in APOE4 BMDMs (Supplementary Fig. S3B). GLUT8, also known as SLC2A8, is a trehalose transporter localized in endosomal or lysosomal membranes, which plays an important role in trehalose transport in primary mouse hepatocytes and HEK293 cells [36–38]. Trehalose was previously shown to stimulate macrophage autophagy and autophagy-lysosomal biogenesis in vitro and in vivo [39]. Moreover, trehalose is a potent autophagy inducer in neurological disorders such as AD and Parkinson’s disease with the ability to cross the blood–brain barrier and enter the central nervous system [40]. Western blot assay showed that GLUT8 was low expressed in APOE4 BMDMs and mouse cochleae (Fig. 5A; Supplementary Fig. S6A, B). Treatment with myelin debris induced an increase of the LC3II/LC3I ratio and downregulation of SQSTM1/P62 in BMDMs, which was more significant in APOE3 BMDMs than in APOE4 BMDMs, indicating that autophagy was damaged in APOE4 BMDMs. In BMDMs treated with myelin debris, co-treatment with trehalose led to an increase in the LC3II/LC3I ratio and upregulation of SQSTM1/P62, which were more significant in APOE4 than in APOE3 BMDMs (Fig. 5B, C), indicating that the impaired lipophagy in APOE4 BMDMs could be partially restored by trehalose treatment.

Immunofluorescent assay showed LC3-positive puncta and LDs were increased in APOE4 and APOE3 BMDMs treated with myelin debris (Fig. 5D). Notably, treatment with myelin debris (+Mye) induced a more significant increase of LDs in APOE4 BMDMs than that in APOE3 BMDMs (Fig. 5E), and meanwhile a more significant increase of LC3-positive puncta was detected in APOE3 BMDMs than that in APOE4 BMDMs (Fig. 5F). In addition, the proportion of LC3-positive LDs was lower in APOE4 BMDMs (+Mye) than in APOE3 BMDMs (+Mye) (Fig. 5G). The subsequent co-treatment with trehalose (+Mye/Tre) led to an increase of LC3-positive puncta in both APOE4 and APOE3 BMDMs (Fig. 5F). However, LDs in APOE4 BMDMs (+Mye/Tre) were more significantly reduced than that in APOE3 BMDMs (+Mye/Tre) (Fig. 5E). These results suggest that trehalose can effectively reduce lipid accumulation by enhancing lipophagy in APOE4 BMDMs.

## Discussion

ARHL are characterised by the damage of ribbon synapse receptors, the reduction of hair cells and neuron damage [7]. In this study, we focused on the role of APOE4 in cochlear neuron damage and reported the presence of demyelination in the cochleae of APOE4 mice, which may be directly related to hearing loss associated with APOE4. The APOE4 allele has been extensively studied for its association with central nervous system damage, particularly in the pathogenesis of AD [2]. Additionally, there is evidence linking APOE4 to peripheral nerve damage, such as that observed in MS [4]. Epidemiological studies have indicated that the presence of one APOE4 allele may increase the risk of ARHL, while the presence of *ApoE-ε4* homozygote is associated with more severe hearing impairment and earlier onset. In addition, APOE4 is reported to induce the damage of cortical areas in the brain related to speech cognition [41].

The abnormal demyelination of neurons is known to be detrimental to the central nervous system [42]. Previous research has revealed the typical phenotypes of MS, including inflammatory and axonal demyelination [43]. Macrophages play a crucial role in the clearance of myelin debris, infectious materials and pathogenic protein aggregates seen in MS, which is essential for cholesterol recycling and the axonal remyelination by the recruitment of oligodendrocyte and Schwann cells [31]. Impaired phagocytosis and defect autophagy in macrophages could reduce the clearance of myelin debris and impede the renewal of myelin sheaths, thereby inducing neuron lesion [19, 21]. This intricate interplay between macrophages and myelin sheaths is a key aspect of the pathophysiology of demyelinating diseases. The present study revealed that APOE4-induced demyelination in cochlear SGNs might have the same pathogenic mechanisms to demyelinating diseases. Given the ongoing process of myelin sheath renewal on the axons of spiral neurons, phagocytosis is a critical mechanism that facilitates macrophages to uptake and degrade cholesterol-rich myelin debris [21]. Our study indicated that APOE4 reduced the phagocytic capacity in macrophages and impair the clearance of myelin debris, which promoted demyelination of SGNs in mouse cochleae and contributed to SGNs lesion.

Literature reports suggest that increased cholesterol metabolism levels after spinal cord injury promote remyelination [44]. APOE is a principal regulator of lipid transport and cholesterol homoeostasis in the central and peripheral nervous system [15]. Compared with APOE3, APOE4 exhibits significant differences in the secretion of cholesterol and phospholipids, lipid binding capacity, and intracellular degradation [16]. These differences may be attributed to the distinct structure of APOE4, where arginine 61 forms an ionic interaction with glutamic acid 255, resulting in a conformation less stable than that of other APOE isoforms. This structural instability may confer inferior efficacy of APOE4 in cholesterol transport and metabolism relative to APOE3 [45]. Based on the KEGG database analysis of APOE3 and APOE4 BMDMs proteomics, APOE4 macrophages may suffer from the disruption of cholesterol homoeostasis, which were consistent with increased intracellular cholesterol and retained LDs in APOE4 macrophages detected by flow cytometry assay and immunofluorescence staining.

The autophagy function of macrophages plays an important role in the clearance of myelin debris, as it allows for the efficient degradation and recycling myelin debris [9]. In this study, the proteomic analysis between APOE4 BMDMs and APOE3 controls showed that most differentially expressed proteins are localised in mitochondria and lysosomes, suggesting a decline of autophagy function in APOE4 macrophages, which were consistent with the findings in RCMs and BMDMs. Given the potential feasibility of enhancing the autophagy level of microglia and macrophages for the treatment of age-related neurodegenerative diseases [46–49], it was expected to explore new strategies to alleviate APOE4-induced ARHL associated with defective autophagy in RCMs.

Proteomic data analysis identified the downregulation of the expression of the trehalose transporter GLUT8 in APOE4 mice. Trehalose, an effective autophagy inducer, has shown therapeutic potential in neurological diseases such as AD and Parkinson’s disease [40, 50]. Previous studies have also shown that trehalose has a reparative effect on the autophagy function of BMDMs [39, 49]. Our study found that the defect autophagy in APOE4 BMDMs could be rescued by the treatment of trehalose, manifesting the enhanced clearance of myelin debris and decreased LDs. The present study suggested that enhancing autophagy in RCMs by trehalose-treatment could be a potential strategy for APOE4-induced ARHL, which would be further investigated in our future work.

**Fig. 6:**
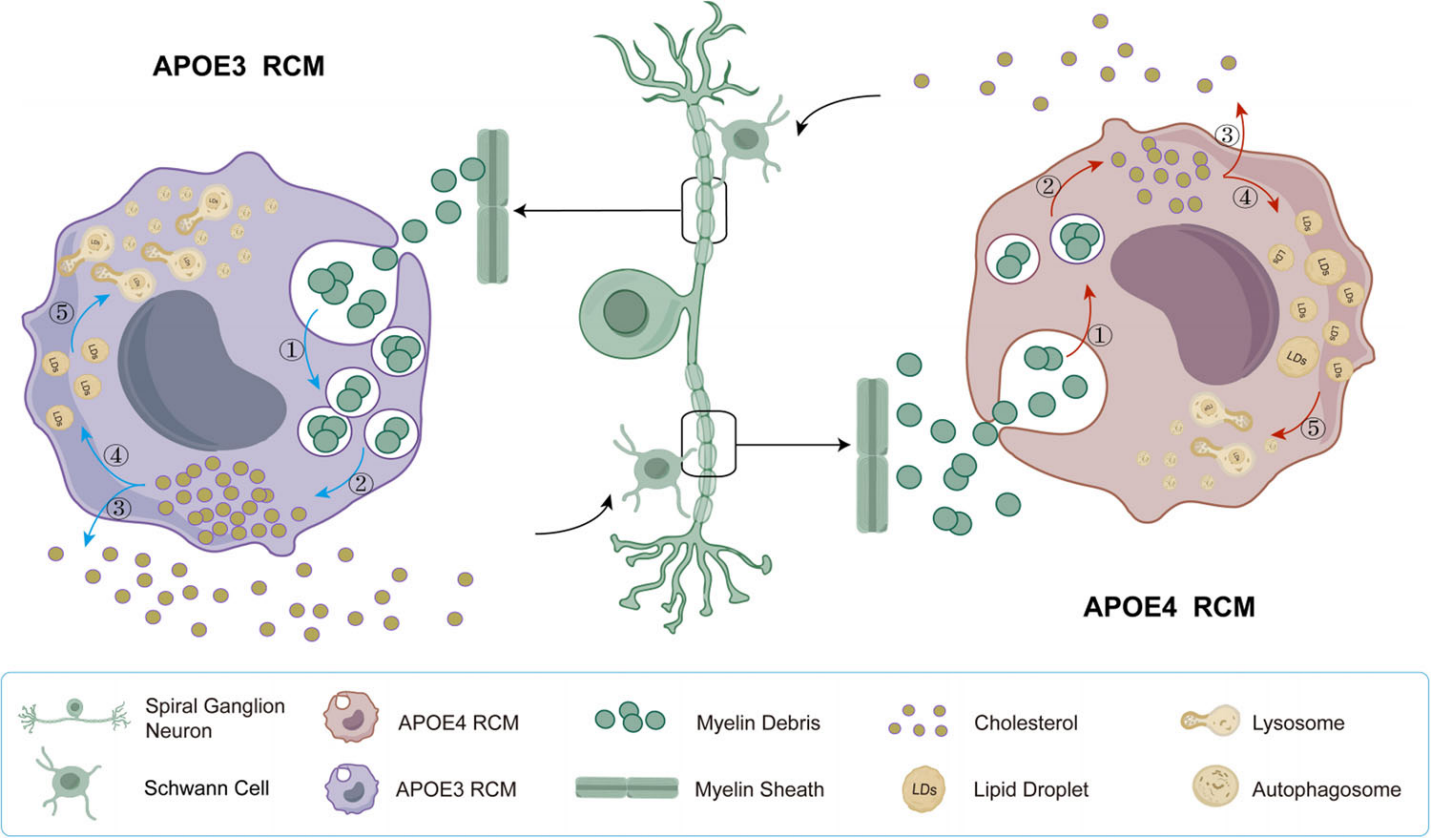
Proposed schematic diagram of APOE3 and APOE4 RCMs in the clearance of myelin debris from demyelinated SGNs. The five-step process of Myelin debris clearance: ① Following axonal demyelination of SGNs, myelin debris is phagocytosed and internalized by RCMs. ② The phagocytosed myelin debris is degraded into cholesterol through the lysosomal degradation pathway. ③ A portion of the cholesterol is effluxed from RCMs and reused by Schwann cells to form new myelin sheaths. ④ The remained intracellular cholesterol is used for LDs synthesis. ⑤ LDs are degraded through the lipophagy pathway. APOE3 RCMs could efficiently phagocytose myelin debris (step ①) and maintain the LDs homoeostasis via step ②-⑤. On the contrary, APOE4 RCMs exhibit impaired phagocytic ability for myelin debris clearance (step ①). In addition, APOE4 RCMs showed a defect lipophagy, resulting in abnormal accumulation of LDs (step ④ and ⑤), which could synthetically impair the myelin debris clearance by RCMs and diminish the reused cholesterol in repairment of demyelinated lesions of SGNs.

This study revealed that APOE4 induced defect phagocytosis and impaired lipophagy in RCMs, which might diminish the clearance of myelin debris by RCMs and promote axonal demyelination in SGNs (Fig. 6). These findings may help to elucidate the pathogenic mechanism of APOE4 in auditory centres and provide a potential therapeutic strategy for ARHL induced by APOE4-implicated neurodegeneration.

## Materials and methods

### Mice

Homozygous APOE4 mice and APOE3 mice used in the experiments were purchased from The Jackson Laboratory. Briefly, for the APOE4 mice, the human APOE allele was inserted at the mouse *ApoE* locus on chromosome 7. The exon 1 of mice remained unchanged and exons 2–4 were replaced with human the *ApoE-ε4* sequence through homology directed repair. APOE3 mice was generated using CRISPR/Cas9-mediated gene targeting in APOE4 zygotes. All mice were purified and bred by the Animal Center of Nanjing Medical University under stringent husbandry conditions (IACUC: 2002003). The indoor temperature was maintained within a range of 20–26 °C, and the humidity was maintained within a range of 40–70%. A 12/12 h light-dark photoperiod was implemented daily. Bedding, feeding, and drinking water were sterilized using high-temperature and high-pressure Co-irradiation to ensure a sterile environment. Bedding was replaced 2–3 times per week to maintain good hygiene.

### Histochemistry staining

Cochleae were dissected from the temporal bone and fixed in 4% paraformaldehyde (PFA) in phosphate buffer saline (PBS) at pH 7.4 and 4 °C overnight. In the study to detect whether APOE4 affects demyelination in mice, using an anti-MBP antibody (Abclonal, China), cochleae from APOE3 and APOE4 10-month-old mice were used, with a sample size of 6 mice for each group. The cochleae were then placed in 10% ethylene diamine tetraacetic acid (EDTA) solution and decalcified for 14 days. Following decalcification, the samples were processed through dehydration, embedding, and sectioning. Paraffin sections were dewaxed with xylene, washed with gradient alcohol and citric acid for antigen retrieval, and then washed in PBS buffer three times for 5 min each. After washing, the sections were incubated in 3% hydrogen peroxide in PBS for 25 min at room temperature to block endogenous peroxidase activity, followed by three washes with PBS for 5 min each. The sections were then blocked with 5% milk goat serum in PBS for 1 h at room temperature to prevent nonspecific antibody binding. The sections were incubated with the primary antibodies overnight at 4 °C, rinsed, and incubated with HRP-conjugated secondary antibodies or phalloidin for 1 h at room temperature. Finally, the sections were developed and stained with diaminobenzidine (Servicebiol, China), washed, and mounted on slides. Slides were dehydrated with increasing concentrations of ethanol followed by xylene and sealed with neutral resin. Slides were scanned using an Olympus BX53 microscope at 20× magnification, and images were analysed using the ImageJ software.

### Transmission electron microscopy (TEM)

Cochleae were extracted from anesthetised mice, with a sample size of three 10-month-old mice for each of the APOE3 and APOE4 genotypes. The cochleae were then fixed in glutaraldehyde at 4 °C overnight, transferred to decalcification solution for 14 days, dissected under a stereomicroscope to remove basilar membrane and SGNs tissue, fixed in fresh glutaraldehyde at 4 °C overnight, and processed for TEM (FEI, Tecnai G2F30, USA). The myelin sheath of SGNs, which normally manifested as a series of compacted layers around the axon [51], were characterized by TEM observation. Myelin g-ratio, defined as the ratio between the inner and the outer diameter of the myelin sheath, was also measured. The axonal demyelination of SGNs were featured by the disrupted continuity and intactness of the myelin sheath with reduced myelin g-ratio [52].

### Treatment of mouse cochlea using demyelinating agent

LPC was used as a demyelinating agent via round-window administration. Briefly, APOE3 and APOE4 10-month-old mice were anesthetised and tympanic membranes were exposed using forceps; 1 μL of 1% LPC (MCE, USA) with Evans Blue as an indicator was injected into the cochleae of the left ears of each mouse to serve as the experimental group, while an equal volume of PBS was injected into the right ears to serve as the control group. Following injection, the mice were allowed to survive for 24 h before being euthanised. The cochleae were then separated and processed for subsequent experiments, including staining procedures as described in sections of histochemistry staining and immunofluorescent staining.

### Myelin fragment extraction

Myelin was purified from post-mortem mouse brain tissue by density-gradient centrifugation as described previously. The brains were removed from mice, stored at −80 °C, and then processed to obtain purified myelin. The myelin was homogenised and stored at −80 °C until further use. Protein concentrations were determined by bicinchoninic acid assay.

### Cell culture and treatment

L929 cells were cultured, and the culture supernatants were collected. BMDMs were extracted and cultured as previously described [53]. BMDMs were cultured in Dulbecco’s modified Eagle medium DMEM supplemented with 10% L929 supernatant, 10% foetal bovine serum, and 1% Penicillin-Streptomycin Solution. After 7 days in culture, adherent BMDMs were harvested and plated at 0.5 × 10^6^ cells/ml for use in in vitro experiments. BMDMs were treated with mouse myelin (100 μg/mL) or 40 μg/mL cholesterol for 24 h. To induce autophagy, cells were treated with 50 mM trehalose for 24 h before collection. To measure autophagic flux, cells were treated with 20 μM chloroquine phosphate for 3 h before adding myelin debris [53, 54].

### Immunofluorescent staining

In the experiments involving cochlear staining following LPC injection, a total of twelve mice were used, with six 10-month-old mice for each of the APOE3 and APOE4 genotypes. Cochleae were dissected and fixed in 4% PFA in PBS (pH 7.4) at 4 °C overnight, then placed in 10% EDTA solution and decalcified for 14 days. The samples were then dehydrated, embedded, and sectioned. Paraffin sections were dewaxed with xylene, washed with gradient alcohol and citric acid for antigen retrieval, and then rinsed in PBS three times for 5 min each. After rinsing, the sections were blocked with 5% milk goat serum in PBS for 1 h at room temperature to prevent nonspecific antibody binding. The sections were then incubated with the primary antibodies overnight at 4 °C, rinsed, and incubated with Alexa Fluor 488/555/647 conjugated secondary antibodies or phalloidin for 1 h at room temperature. The following primary antibodies were used: MBP (Abcam, USA), LC3 (CST, USA), SQSTM1/P62 (CST, USA), LAMP1 (Abcam, USA), CD36 (Abcam, USA), and F4/80 (Abcam, USA). Subsequently, the nuclei were counterstained with DAPI. Sections were imaged using confocal microscopy or an Olympus BX53 microscope.

For the staining of BMDMs on cell culture slides, a sample size of 6 mice for each of the APOE3 and APOE4 genotypes at 6–8 weeks of age was utilised. The BMDMs were fixed with 4% PFA for 15 min at room temperature, permeabilised with 0.1% Triton X-100, and blocked in PBS containing 5% goat serum. After incubation in blocks containing primary antibodies for 2 h at room temperature or overnight at 4 °C, the cells were incubated with appropriate secondary antibodies for 1 h at room temperature. If utilising BODIPY staining, during this step, cells should be incubated with the appropriate secondary antibodies conjugated with a 1:1000 dilution of BODIPY dye (Thermo Fisher Scientific, USA). Subsequently, the nuclei were counterstained with DAPI. The samples were visualised with a Zeiss LSM 700 confocal microscope. The acquired images were processed and analysed using ZEN2 software (Carl Zeiss).

### Western blot

Protein extraction from cochleae and BMDMs was performed using distinct procedures. For cochlear tissue, the cochleae were isolated from a total of 12, 10-month-old mice, with 6 mice for each of the APOE3 and APOE4 genotypes. The cochleae were then mechanically ground using an automatic grinder. In the case of BMDMs, the cells were derived from 12 mice aged 6–8 weeks, with 6 mice for each genotype. The cells were treated with trypsin in the culture dish to facilitate digestion, then terminated by centrifugation, and the supernatant was discarded. RIPA buffer was then added to lyse the cells and extract the protein. The lysate was sonicated using an ultrasonic device to shear DNA and shear stress. The protein samples were subsequently mixed with loading buffer and heated at 100 °C for 10 min to denature the proteins.

The protein concentration in each sample was measured using bicinchoninic acid assay. Volumes corresponding to 10 μg of protein for each sample were loaded into wells, and a current of 80 V was applied for 2 h. The gel was transferred to a membrane for 45 min, blocked in 5% milk in TBST for 2 h at room temperature, and incubated with primary antibody overnight at 4 °C. The primary antibodies used in this western blot experiment included those specific for MBP, LC3, SQSTM1/p62, GLUT8, SREBP-1, and SREBP-2. After incubation with the primary antibodies, the membranes were washed with TBST three times to remove unbound antibodies. They were then incubated with HRP-conjugated secondary antibodies (diluted at 1:5000) for 2 h at room temperature followed by the application of chemiluminescence activator to visualize the protein bands.

### Quantitative real-time PCR (qRT-PCR)

The cochlear tissues were collected from a total of 12, 10-month-old mice, with 6 mice for each of the APOE3 and APOE4 genotypes. For BMDMs, the sample size consisted of 12 mice aged 6–8 weeks, with 6 mice for each genotype. Total RNA from mouse cochleae and BMDMs was extracted following TRIzol Kit (Invitrogen, USA) instructions. Then, 1 μg of total RNA was used for cDNA synthesis with a HiScript II One Step qRT-PCR Kit (Vazyme, China). qRT-PCR was conducted on a StepOne Plus system (Applied Biosystems, USA) with ChamQ SYBR qPCR Master Mix (Vazyme, China). To analyse the relative expression levels of the chosen genes, the comparative CT method (2^−ΔΔCT^) was employed. Each qRT–PCR analysis was repeated at least three times, and GAPDH served as an internal control for consistent comparison. The primers used to amplify target genes by qRT–PCR were as follows: Gapdh F (5′-AGGTCGGTGTGAACGGATTTG-3′) and R (5′-TGTAGACCATGTAGTTGAGGTCA-3′), Mbp F (5′-GGCGGTGACAGACTCCAAG-3′) and R (5′-GAAGCTCGTCGGACTCTGAG-3′), and Slc2a8 F (5′-AAGTTCAAGGCTTTGCGGTG-3′) and R (5′-TTGGTGAGGACACAGATGCC-3′)

### Flow cytometry

The cochlear tissues were collected from a total of 20, 10-month-old mice, with 10 mice for each of the APOE3 and APOE4 genotypes. For BMDMs, the sample size consisted of six mice aged 6–8 weeks, with three mice for each genotype. Tissues were detached using 0.25% trypsin without EDTA. Cells were collected, suspended in flow cytometry wash solution, stained with fixable viability dyes, then washed three times in 500 μL of stain buffer. Then, the cells were incubated with 5 μg/mL of CD45 monoclonal antibody conjugated with BV510, 2 μg/mL of CD11b monoclonal antibody conjugated with PE, 10 μg/mL of Filipin-III conjugated with BV421, and 5 μg/mL of F4/80 conjugated with BV510 for 30 min on the ice. After washing, the samples were analysed by flow cytometry.

### Proteomic analysis

In proteomic studies, BMDMs were extracted from a total of six mice, with three 6- to 8-week-old mice for each of the APOE3 and APOE4 genotypes, and cultured for 7 days to induce differentiation prior to experimentation. Following the method described in western blot for extraction and quality control, proteins were treated with 5 mM DTT (Amresco, USA) and incubated at 37 °C for 1 h. Subsequently, 10 mM iodoacetamide was added for 45 min at room temperature in the dark. The sample was diluted 4-fold with 25 mM ammonium bicarbonate (Sigma-Aldrich, USA), and trypsin (Promega, China) was introduced at a 50:1 protein-to-trypsin ratio, with incubation proceeding at 37 °C overnight. On the second day, formic acid (Sigma-Aldrich, USA) was added to reduce the pH below 3, halting enzymatic digestion. The sample underwent desalting via a C18 column, activated with 100% acetonitrile (J.T.Baker, USA) and equilibrated with 0.1% formic acid (Sigma-Aldrich, USA). After loading, washing with 0.1% formic acid removed impurities, and elution with 70% acetonitrile was completed. The flow-through was collected and the sample was lyophilised.

The lyophilised powder was reconstituted in 10 µL of Solution A (100% water, 0.1% formic acid) and centrifuged at 14,000 × *g* and 4 °C for 20 min. A 1 µg sample of the supernatant was injected for liquid chromatography-mass spectrometry analysis. The liquid chromatography elution conditions are shown in Supplementary Table S1.

An ORBITRAP ECLIPSE mass spectrometer, equipped with a FAIMS Pro™ Interface, was utilised with a compensation voltage alternating between −45 and −65 every 1 s. The Nanospray Flex™ ion source operated at an ion spray voltage of 2.0 kV, with the ion transfer tube temperature set to 320 °C. The data-dependent acquisition mode was employed, with scanning m/z of 350–1500 and a primary resolution of 120,000 (at 200 m/z); the AGC target was 4 × 10^5^, and the C-trap maximum injection time was 246 ms. Secondary detection in “Top Speed” mode achieved a resolution of 15,000 (at 200 m/z), with an AGC target of 5 × 10^4^ and maximum injection time of 22 ms. Peptide fragmentation collision energy was set at 33%, yielding raw mass spectrometry data (.raw).

The Mus musculus database was selected for the study, adhering to principles based on species sequencing, annotation completeness, and sequence reliability. The search was conducted using Proteome Discoverer 2.4 software (Supplementary Table S2).

### Statistical analysis

For the analysis of images detected by TEM, immunofluorescent staining and western blot, positive signals were quantified using ImageJ. Subsequent statistical analysis of the quantified data was performed using GraphPad Prism 9. For ELISA and qRT-PCR, direct statistical analysis was conducted using GraphPad Prism 9. In the case of LD analysis, the OriginLab 8.5 software was utilised for the statistical evaluation. All figure legends were meticulously compiled to include pertinent information regarding the statistical tests employed and sample sizes analysed, ensuring transparency and reproducibility of the results.

## Supplementary information

Supplementary Materials

Original Data (WB)

## Data Availability

The mass spectrometry proteomics data have been deposited to the ProteomeXchange Consortium (https://proteomecentral.proteomexchange.org) via the iProX partner repository with the dataset identifier PXD059706.
