# Supplementary material for: APOE4 impairs macrophage lipophagy and promotes demyelination of spiral ganglion neurons in mouse cochleae
Source: Cell Death Discov. 2025 Apr 21;11:190. doi: 10.1038/s41420-025-02454-4 (PMC12012174; doi:10.1038/s41420-025-02454-4)
Supplement: Supplementary file 1 — Supplementary Materials [file 41420_2025_2454_MOESM1_ESM.docx]

**APOE4 impairs macrophage lipophagy and promotes demyelination of spiral ganglion neurons in mouse cochleae**

**Junru Chen^1^, Haibing Chen^1, 2^, Qinjun Wei^1, 3^, Yajie Lu^1, 3^, Tianming Wang^4^, Xiuhong Pang^5^, Guangqian Xing^2^, Zhibin Chen^2*^, Xin Cao^1, 3*^, Jun Yao^1, 3, 5*^**

^1^Department of Medical Genetics, School of Basic Medical Science, Nanjing Medical University, Nanjing, China

^2^Department of Otolaryngology, the First Affiliated Hospital with Nanjing Medical University, Nanjing, China

^3^Jiangsu Key Laboratory of Xenotransplantation, Nanjing Medical University, Nanjing, China

^4^Central Laboratory, Translational Medicine Research Center, the affiliated Jiangning Hospital of Nanjing Medical University, Nanjing, China

^5^Department of Otolaryngology-Head and Neck Surgery, the Affiliated Taizhou People's Hospital of Nanjing Medical University, Taizhou School of Clinical Medicine, Nanjing Medical University, Taizhou, China

^*^**Corresponding author**: Zhibin Chen

Department of Otolaryngology, the First Affiliated Hospital with Nanjing Medical University

No.300 Guangzhou Road, Nanjing, China 210029

E-mail address: czbnj@163.com (Z. Chen)

^*^**Corresponding author**: Xin Cao

Department of Medical Genetics, School of Basic Medical Science, Nanjing Medical University

No.101 Longmian Avenue, Nanjing, China 211166

Tel/Fax: +86-25-86869341

E-mail address: caoxin@njmu.edu.cn (X. Cao)

^*^**Corresponding author**: Jun Yao

Department of Medical Genetics, School of Basic Medical Science, Nanjing Medical University

No.101 Longmian Avenue, Nanjing, China 211166

Tel/Fax: +86-25-86869413

E-mail address: [joelyao@njmu.edu.cn](mailto:joelyao@njmu.edu.cn) (J. Yao)

**Supplementary Materials**

**Supplementary Tables:**

**Table 1. Liquid chromatography elution conditions in Proteomic analysis**

| Time (min) | Ratio of mobile phase B (%) |
| --- | --- |
| 0 | 6 |
| 8 | 6 |
| 16 | 12 |
| 71 | 30 |
| 83 | 40 |
| 84 | 95 |
| 94 | 95 |
| 95 | 6 |

**Table 2. Proteome Discoverer 2.4 software parameters in Proteomic analysis**

| Parameter | Value |
| --- | --- |
| Enzyme | Trypsin |
| Static modification | Carbamidomethyl (C) |
| Dynamic modification | M Oxidation (15.995Da): Acetyl (Protein N-terminal) |
| Precursor ion mass tolerance | ± 15 ppm |
| Fragment ion mass tolerance | ± 0.02 Da |
| Max missed cleavages | 2 |

**Supplementary Figures:**


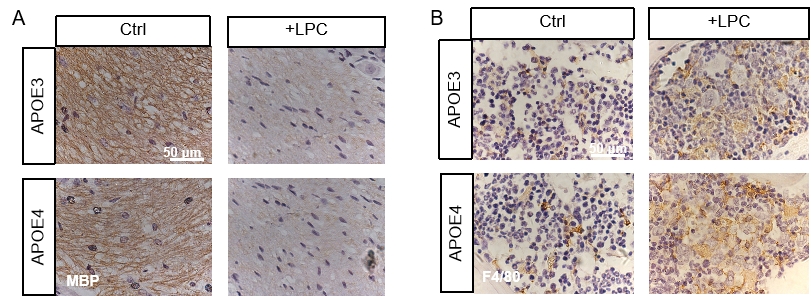


**Fig. S1** Immunohistochemical assay of cochlear spiral ganglion in APOE4 and APOE3 mice. **A** Immunohistochemical staining of MBP in cochlear spiral ganglion of 10-month-old APOE4 and APOE3 mice treated with or without LPC. Scale bar: 50 μm. **B** Immunohistochemical staining of F4/80 in cochlear spiral ganglion of 10-month-old APOE4 and APOE3 mice treated with or without LPC. Scale bar: 50 μm.


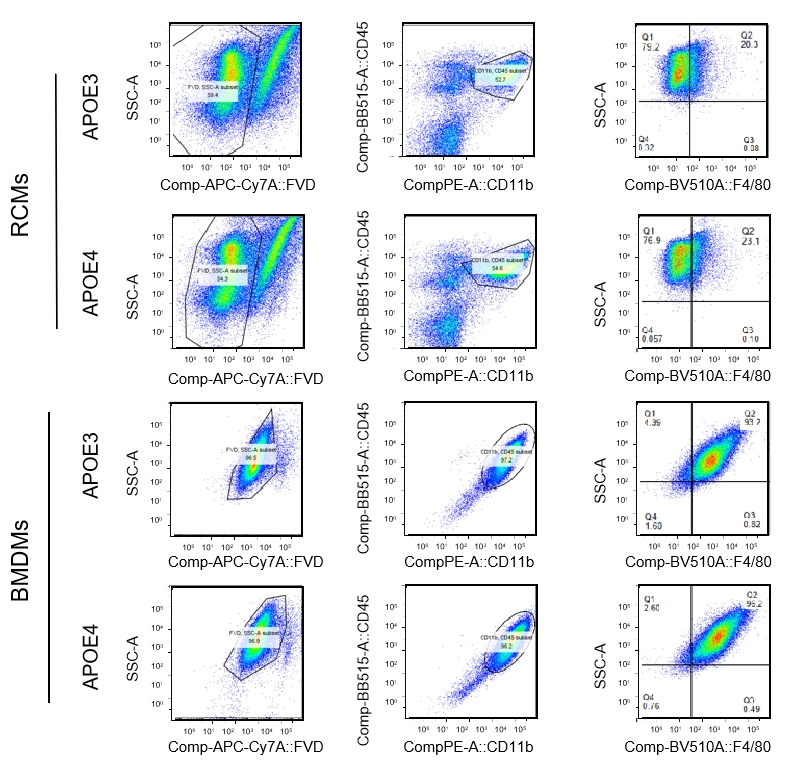


**Fig. S2** Flow cytometry assay of RCMs and BMDMs stained with CD45 (APC-Cy7A), CD11b and F4/80 (n = 3). The results showed that protein typing of RCMs (CD45^high^, CD11b^high^) were homologous to that of BMDMs.

**
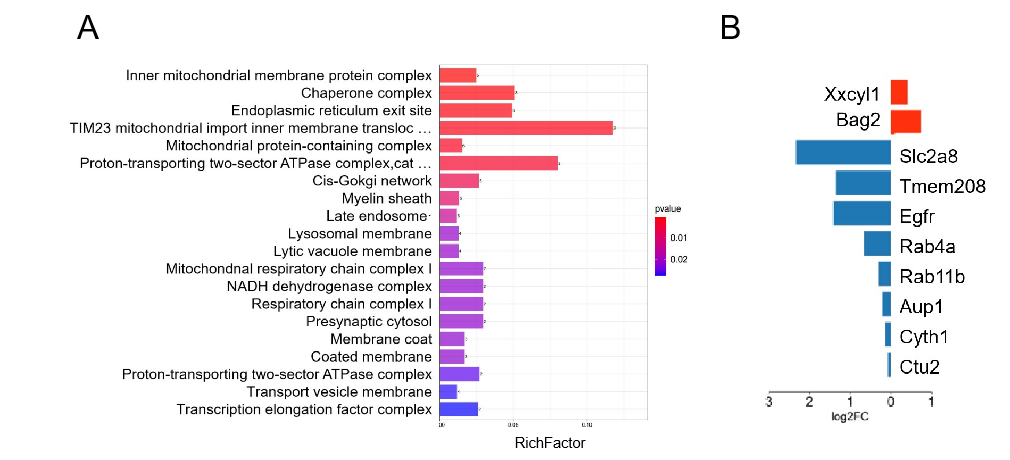
**

**Fig. S3** Proteomic GO analysis of APOE3 and APOE4 BMDMs. **A** GO analysis of proteomics in APOE3 and APOE4 BMDMs. **B** Autophagy-related proteins differentially expressed in APOE3 and APOE4 BMDMs (n = 3, *P* < 0.1).


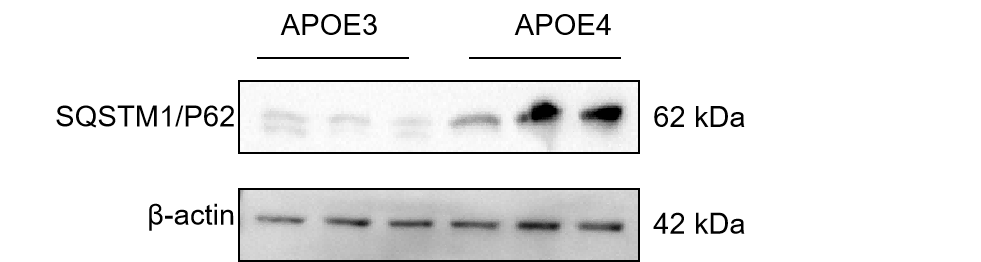


**Fig. S4** Western blot of SQSTM1/P62 in the cochleae of 10-month-old APOE4 and APOE3 mice (n = 3).


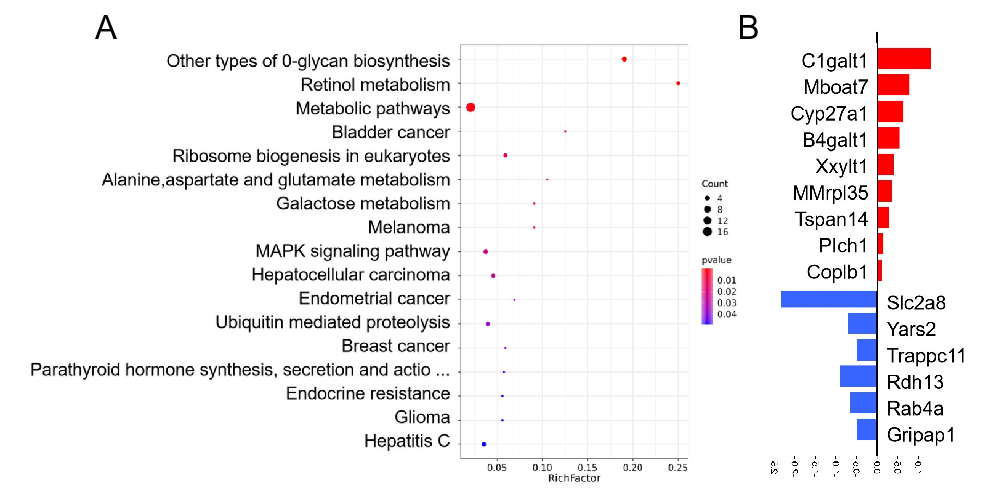


**Fig. S5** Proteomic KEGG analysis of APOE3 and APOE4 BMDMs. **A** KEGG analysis of proteomics in APOE3 and APOE4 BMDMs. **B** Cholesterol- related proteins differentially expressed between APOE3 and APOE4 BMDMs (n = 3, *P* < 0.1).


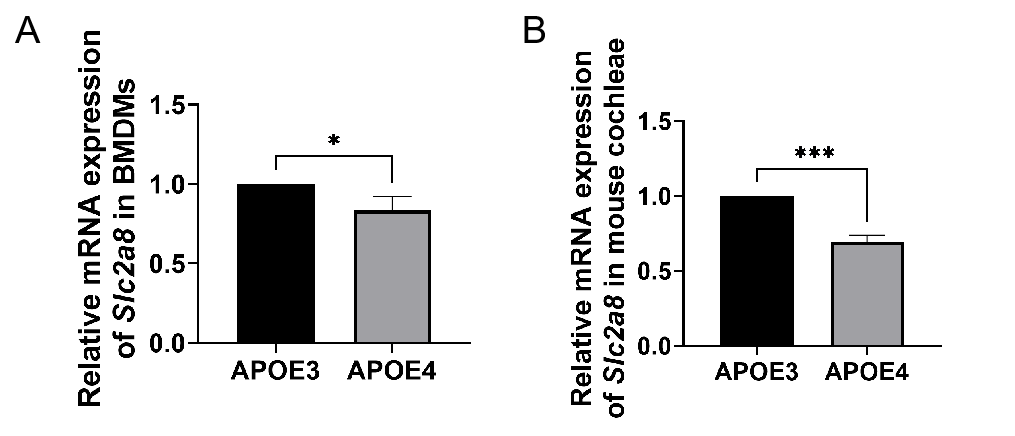


**Fig. S6** Relative mRNA expression of *Slc2a8* in BMDMs (A) and cochleae of 10-months-old APOE4 and APOE3 mice (B). n = 3, **P* < 0.05, ****P* < 0.001 by one-way ANOVA).
