# Supplementary material for: APOE4 impairs macrophage lipophagy and promotes demyelination of spiral ganglion neurons in mouse cochleae
Source: Cell Death Discov. 2025 Apr 21;11:190. doi: 10.1038/s41420-025-02454-4 (PMC12012174; doi:10.1038/s41420-025-02454-4)
Supplement: Supplementary file 2 — Original Data (WB) [file 41420_2025_2454_MOESM2_ESM.docx]

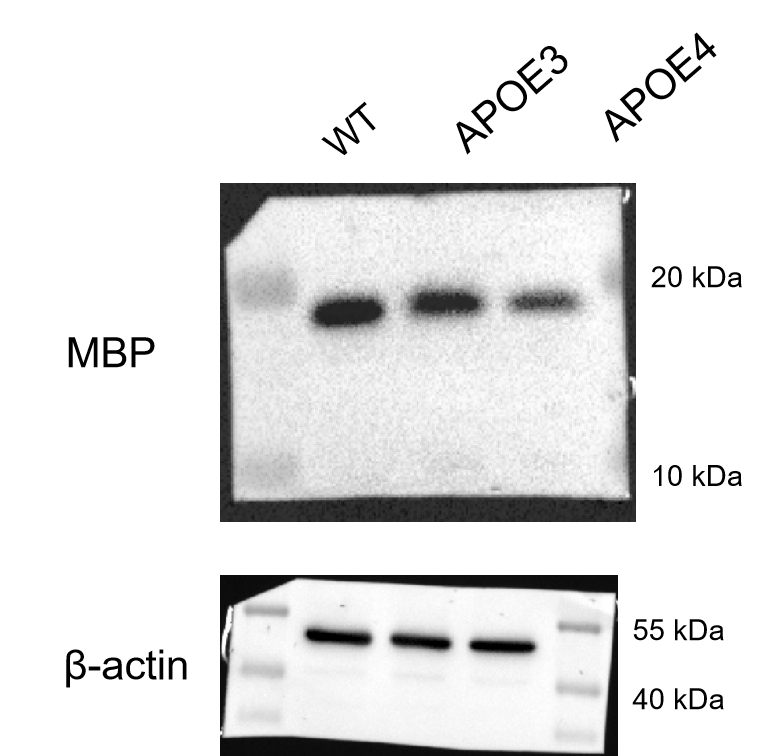


Original western blot in Fig. 1D


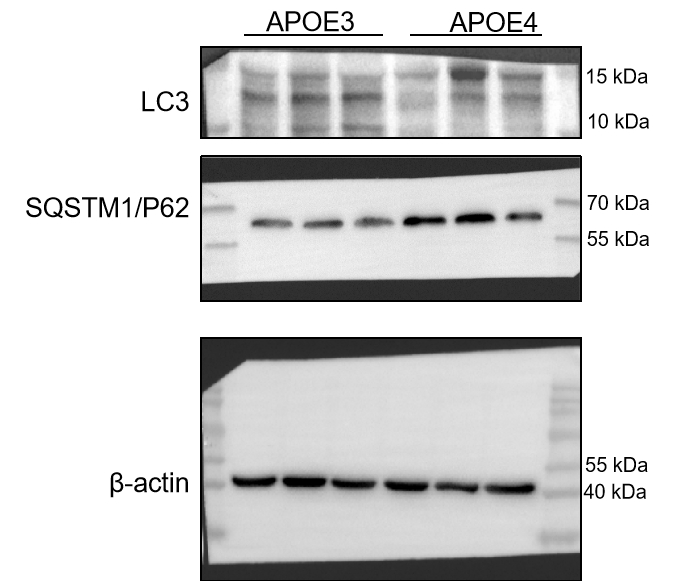


Original western blot in Fig. 3F


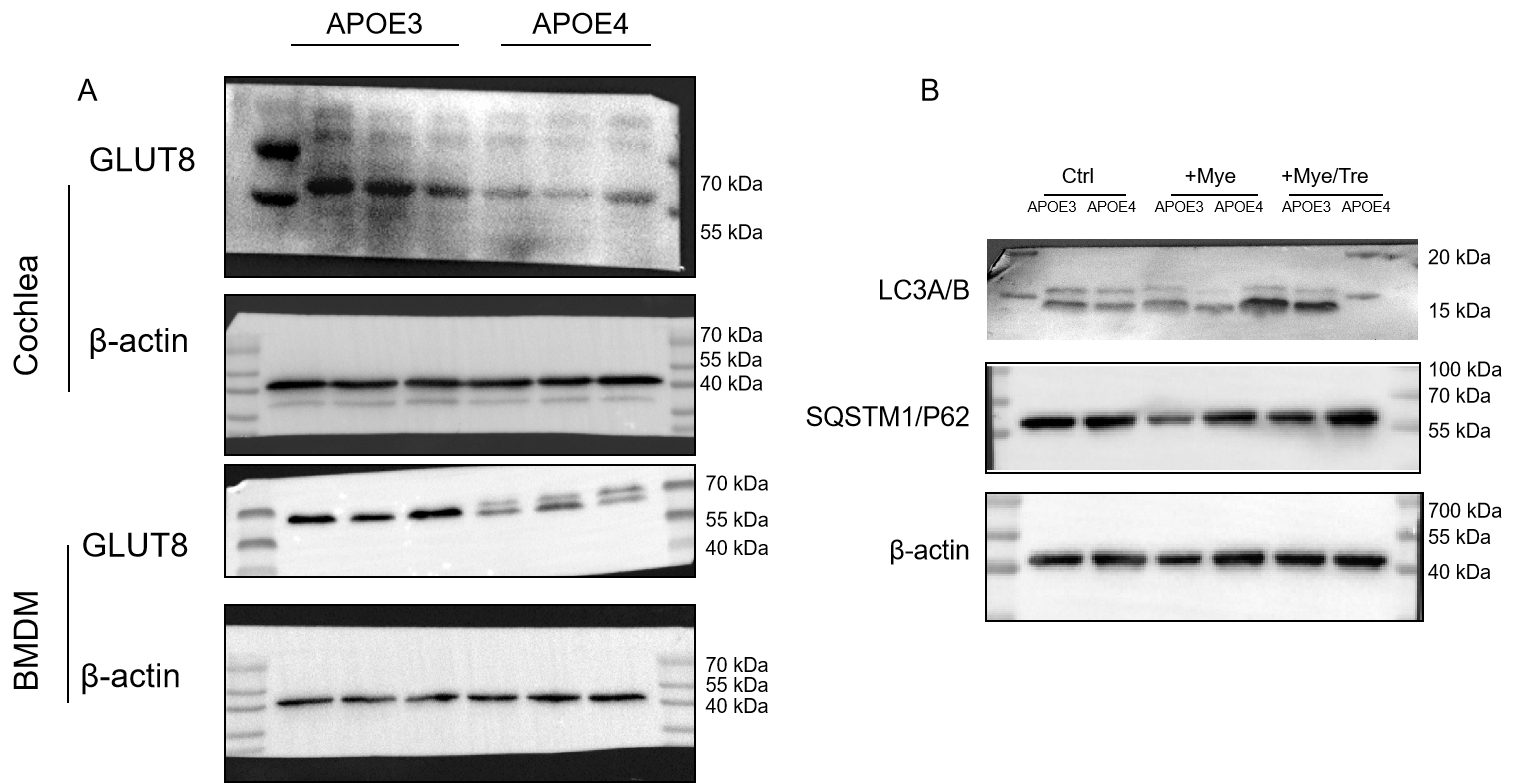


Original western blot in Fig. 5A, B


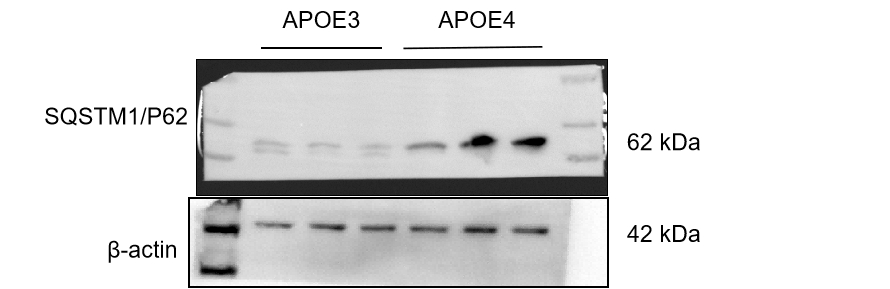


Original western blot in Fig. S4
